# Neonatal Host Defense against Staphylococcal Infections

**DOI:** 10.1155/2013/826303

**Published:** 2013-07-10

**Authors:** Melanie R. Power Coombs, Kenny Kronforst, Ofer Levy

**Affiliations:** ^1^Pathology, Dalhousie University, Halifax, NS, Canada B3H 4R2; ^2^Lurie Children's Hospital Chicago, IL 60611, USA; ^3^Feinberg School of Medicine, Northwestern University, Chicago, IL 60611, USA; ^4^Division of Infectious Diseases, Boston Children's Hospital, 300 Longwood Avenue, Boston, MA 02115, USA; ^5^Human Biology & Translational Medicine, Harvard Medical School, Boston, MA, USA

## Abstract

Preterm infants are especially susceptible to late-onset sepsis that is often due to Gram-positive bacterial infections resulting in substantial morbidity and mortality. Herein, we will describe neonatal innate immunity to *Staphylococcus* spp. comparing differences between preterm and full-term newborns with adults. Newborn innate immunity is distinct demonstrating diminished skin integrity, impaired Th1-polarizing responses, low complement levels, and diminished expression of plasma antimicrobial proteins and peptides, especially in preterm newborns. Characterization of distinct aspects of the neonatal immune response is defining novel approaches to enhance host defense to prevent and/or treat staphylococcal infection in this vulnerable population.

## 1. Introduction

Over 30% of deaths in children under the age of 5 occur within the first 4 weeks of life [1]. In this context, understanding the immunologic mechanisms underlying neonatal susceptibility is essential for the development of novel approaches to prevent and/or treat bacterial infection. Newborns in neonatal intensive care units (NICUs) are at risk of infection. An improvement in practices regarding hand washing, nutrition, skin, and respiratory care decreases *Staphylococcus *spp. infections [2, 3]. Such NICU quality improvements also significantly decrease costs per patient [4]. Antibiotics are the primary treatment for staphylococcal infections, but the use of these agents is also associated with resistance and alteration of the host microbial flora. Herein, we review innate host defense against *Staphylococcus *spp. with an emphasis on *S. epidermidis* (SE) and *S. aureus* (SA). Accordingly, we searched PubMed, a computer-based literature search engine, using the following terms: “newborn” OR “neonate” OR “neonatal” AND “Staphylococcus” AND/OR “sepsis” OR “innate” OR “cytokine” OR “Toll-like receptor” OR “pattern recognition receptor” OR “antimicrobial peptide” OR “neutrophil” OR “monocyte.” We then organized the resulting references grouping them into detector function, effector function, and translational efforts. 

## 2. Neonatal Staphylococci Infections

SE colonizes newborns [5] and remains a part of the human normal flora [6, 7]. SE-induced sepsis in preterm newborns has been associated with an increased risk of adverse common outcomes, prolonged hospital stay, and increased costs [8–17]. SE is the most frequently isolated strain of coagulase-negative staphylococci (CoNS) and is identified diagnostically from SA because of its inability to produce coagulase [18, 19]. SE forms a biofilm on catheters and commonly infects immunocompromised patients [6, 20–22].

Invasive infections due to extracellular pathogens such as CoNS are largely restricted to premature infants. At the University Hospital of Patras in Greece, between 2006 and 2007, 8.5% of all NICU admissions had late-onset CoNS sepsis. SE was the most prevalent organism found, and biofilm production was identified as a determinant for persistent infection [23]. The majority of late-onset sepses (defined as 1 or more positive blood cultures obtained after 72 h of life) in very low birth weight (VLBW) newborns were caused by Gram-positive organisms [19, 24–26]. CoNS were responsible for 48% of infections in a study that examined over 6000 NICU infants in the USA [24]. Risk factors identified included low gestational age, low birth weight, an increased duration of ventilator support, central vascular catheter, and prolonged parenteral nutrition [24]. Close to half of the newborns that were <25 weeks of gestation developed late-onset sepsis and had a longer length of hospital stay [24]. Although CoNS infections often rapidly resolve with a few days of intravenous antibiotics, they are responsible for significant healthcare costs and also induce inflammatory responses that can sometimes result in long-term harm to the newborn, including potential cerebral injury [8–13, 27]. 

SA is the second leading cause of late onset sepsis in neonates [24]. SA leads to more prolonged bacteremia, dissemination to additional anatomic sites (e.g., osteomyelitis), and substantially more sepsis-related deaths than CoNS infections [28, 29]. SA-associated neonatal sepsis is associated with increased antibiotic resistance [28, 30, 31]. Newborns are often colonized with SA from their mothers via horizontal rather than a vertical transfer [32]. Accordingly, a study examining over 400 mothers admitted for preterm labor and the newborns subsequently admitted to the NICU in the USA found that vertical transmission of methicillin-resistant SA (MRSA) at the time of delivery was unlikely [33]. These findings suggested that there was a horizontal transfer of MRSA from health care workers or from parents while taking care of their infants [33]. Indeed, community-based MRSA strains have been identified in some NICU infections in the USA [28]. 

## 3. Innate Immune System in Neonates

Given the “in-born” nature of the innate immune response, it has been surprising that the innate immune response actually develops with age [34]. As has been recently reviewed, the innate immune response in neonates is distinct from that of older individuals [35, 36]. Multiple cells mediate innate immune responses, including skin and mucosal epithelia, neutrophils, monocytes/macrophages, and dendritic cells [35, 36]. The innate immune system also influences the adaptive immune response, and therefore understanding neonatal innate immunity may also inform development of age-specific vaccines. 

### 3.1. Soluble Factors That Modulate Neonatal Immune Responses

Newborn plasma contains multiple factors that modulate the immune response [37]. Neonatal cord blood plasma has significantly more adenosine, an endogenous purine metabolite that inhibits Toll-like receptor (TLR)-mediated Th1 responses, than adult plasma [38]. The neonatal adenosine system inhibits TLR2-induced tumor necrosis factor (TNF) production but not interleukin (IL)-6 [38]. Serum of human newborns in the first week of life demonstrates a higher basal IL-6/TNF ratio than that of adults [39]. Moreover, when compared to monocytes of adults, neonatal cord blood monocytes produce a high ratio of IL-6 to TNF in response to TLR stimulation [39]. IL-6 can impair neutrophil production, migration, and function during sepsis [39–42] possibly contributing to the susceptibility of newborns to bacterial infection. 

### 3.2. Antimicrobial Proteins and Peptides

A key mechanism by which the innate immune systems kill microbes and neutralize microbial toxins is via expression and mobilization of antimicrobial proteins and peptides (APPs) [43–45]. APPs are typically cationic molecules that have membrane-active effects on bacteria. Some APPs have additional function such as lactoferrin, which binds iron, a key nutrient for many bacteria [46], and lysozyme, which has enzymatic activity by muramidase that damages bacterial cell walls [47]. Defensins are small cationic antimicrobial peptides produced by leukocytes and epithelial cells in humans [48, 49]. Of note, preterm human neonates demonstrate deficient expression of plasma APPs that may contribute to the ability of bacteria to proliferate rapidly in preterm bloodstream. Moreover, newborn neutrophils demonstrate impairment in production of nucleic acid-based neutrophil extracellular traps (NETs) that serve as scaffolds for APPs and are important for host defense [50]. Overall, reduced plasma levels of complement and APPs as well as impaired deployment of APPs on NETs may, in part, explain why neonates are more susceptible to infection [51, 52].

### 3.3. Quantitative Differences in Phagocytes

Premature neonates admitted to the NICU have a relatively high frequency of neutropenia that can reach up to 8% [53]. In full-term newborns, impaired function of phagocytes has been described at birth [54]. Newborn neutrophils demonstrate impaired chemotaxis, phagocytosis, and impaired respiratory burst [54–57] and an impaired ability to form extracellular traps important for capture and killing extracellular bacteria [50].

### 3.4. Qualitative Differences in Leukocytes

The neonatal immune response is skewed towards Th2 and anti-inflammatory cytokine production. This may be important for protection of the fetus in utero and to avoid excessive inflammation during colonization with normal flora during the first days of life. Preterm newborns demonstrate mostly an anti-inflammatory response characterized by high IL-10 production while production of other cytokines is relatively low [58].

Inhibitory immune receptors antagonize cell-activating signals. Several of these inhibitory immune receptors function through immunoreceptor tyrosine-based inhibitory motifs (ITIMs). Newborn immune cells express a distinct pattern of inhibitory receptors compared to adult immune cells. Cord blood and 1-month-old newborn neutrophils express higher levels of the inhibitory receptors, leukocyte-associated immunoglobulin- (Ig-) like receptor-1 (LAIR-1), and siglec-9 than adults [59]. However, cord blood monocytes exhibited decreased expression of the immune receptor expressed on myeloid cells (IREM)-1, and 1-month-old newborn monocytes expressed lower levels of LAIR-1 compared to adults [59]. These observations suggest that neonatal neutrophils and monocytes are at a different basal set point from adult leukocytes.

## 4. Toll-Like Receptors (TLRs)

TLRs are pattern-recognition receptors (PRRs) of the innate immune system essential for early recognition of pathogen and also guide the adaptive immune response. There have been 10 TLRs identified in humans that signal through adaptor molecules such as myeloid differentiation factor-88 (MyD88) to activate transcription of immune mediators such as cytokines that direct the response to infection [60, 61]. While basal expression of TLRs is similar on full-term human newborn and adult monocytes [62–64], it can change with gestational age. Extremely low birth weight newborns (ELBW), <28 weeks of gestation, demonstrated lower expression of innate immune receptors TLR2, TLR4, CD14, and MD-2 on neutrophils [65]. Monocyte TLR4 mRNA and protein expression increase with gestational age [66]. In contrast, TLR2 expression is constitutively expressed on monocytes across gestational age and is therefore at similar levels in monocytes of preterms, full-term neonates, and adult monocytes [64]. Interestingly, Gram-positive bacteremia apparently induces increases in neonatal peripheral blood monocyte and granulocyte TLR2 expression in infected human newborns [67, 68]. 

**Figure 1:**
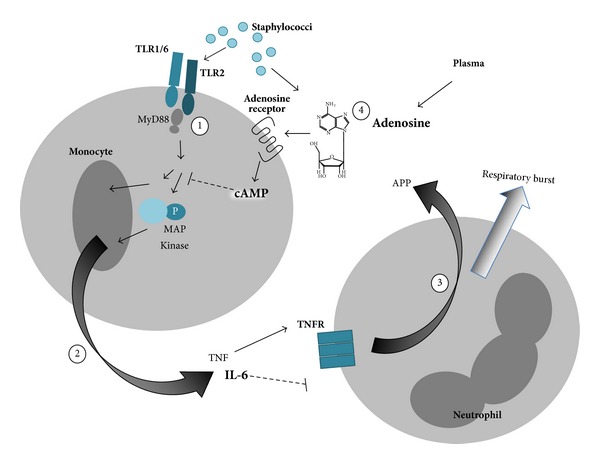
Sensor and effector function of neonatal innate immune system to *Staphylococci *spp. (1) Monocytes detect Staphylococci through TLR2 and (2) result in TLR-mediated production of proinflammatory cytokines such as TNF, and (3) TNF activates neutrophils to produce oxygen radicals and release APPs. (4) Both endogenous plasma and *Staphylococcus*-derived adenosine inhibit pro-inflammatory innate immune responses. Newborn monocytes are deficient in MyD88, activation of MAP kinases, and in TLR-mediated TNF but do produce robust amounts of IL-6, a proresolution cytokine that inhibits neutrophil migration. Overall, this pattern of response impairs neutrophil activation and migration and secretion of APPs. Deficiencies in neonatal responses to staphylococci are depicted by smaller font size, whereas agents that are elevated in newborns are indicated with a larger and bolded font.

Protein expression of MyD88, a cytosolic adaptor molecule essential TLR signaling, was decreased in newborn cord blood neutrophils [69] and monocytes [70] compared to those of adults. MyD88 mRNA levels increase in preterm infants cord blood mononuclear cells along gestational age. Preterm infants demonstrate lower MyD88 mRNA levels, but term infants are comparable to adults [64]; see Figure 1. Thus, there may be an inherent defect in newborns ability to make cytokine in response to infection due to a deficiency in this important signaling molecule. 

A longitudinal study that examined TLR responses of individuals from birth to 2 years of age suggests that there is not a linear progression from an “immature” to “mature” innate immune response from newborns to adults [71]. The percentage of blood monocytes was higher in adults and newborns than 1- and 2-year-olds [71]. 2-year-olds demonstrated greater PAM_3_CSK_4_-(TLR2/1 agonist-) induced levels of intracellular cytokines than adults [71]. There was a higher percentage of 1- and 2-year-old classical (c)DCs making cytokine than adult cDCs [71]. Cytokines secreted from monocytes increased from birth to 2 years old for TNF and IL-1*β*; however, IL-6, IL-23, and IL-10 secretion decreased [71]. Preterm infants cord blood mononuclear cells have a significant defect in IL-12/IL-23p40 production in comparison to term infants after stimulation with TLR2/1 agonist PAM_3_CSK_4_, TLR2/6 agonist Fibroblast-stimulating lipopeptide (FSL), and TLR4 agonist LPS [58].

Micro- (mi-)RNAs involved in inhibiting the TLR4 signaling pathway are increased in newborn monocytes compared to adults and may contribute to decreased cytokine production [72]. Further investigation into the role miRNAs play into TLR2-signaling is warranted to gain further understanding of the potential role of miRNA in the neonatal innate immune response. Further research into other pattern-recognition receptors such as the NOD-like receptors and regulation of those receptors is warranted in the newborn to further understand neonatal staphylococcal-induced sepsis.

## 5. Staphylococcal Infections and Neonatal Host Immune Responses

TLR2 mediates innate immune responses to SE and is essential for clearance of SE in mice [73]. TLR2 also mediates the innate immune response to SA infection [74, 75]. Pretreatment of microglial cells with a TLR2 agonist decreased the inflammatory response to *S. aureus* but enhanced the microglial phagocytosis of this bacterium. Thus, TLR-modulation may be a useful treatment strategy to minimize inflammation in the eye [76].

When interpreting the literature of *in vitro* responses to staphylococci it is important to note that the immune response varies accordingly to whether the bacteria are heat-killed, ethanol-killed, or live [77]. Live SE induced significantly higher levels of cytokines compared to killed SE, including robust activation of the inflammasome for IL-1*β* production, induction of type I interferon production, nuclear factor (NF)*κ*B, and signal transducers and activators of transcription (STAT)1 activation. In contrast, killed SE activated NF*κ*B but did not activate the other innate immune pathways [77]. 

In a novel model of intrajugular infection in mice less than 24 hours of life, newborn mice demonstrate impaired weight gain when injected intravenously with SE compared to saline-injected controls [78]. Similar to the pattern noted in the peripheral blood mononuclear cells of preterm human newborns during Gram-positive bacteremia [67, 68], TLR2 and MyD88 mRNA levels in the liver were significantly increased by injection of SE that induced inocula-dependent serum IL-6 and TNF concentrations [78]. 

SE-induced cytokine production from human neonatal mononuclear cells (MCs) *in vitro* is dependent on gestational age [79–82]. Monocytes of preterm newborns demonstrate reduced IL-1*β*, IL-6, IL-8, and TNF production in response to SE despite adult-level TLR2 expression [83]. Impaired TNF production may contribute to impaired neutrophil responses to *Staphylococcus *spp. as TNF activates neutrophils. SE-induced phosphorylation of cell-signaling molecules (e.g., phospho-p65, phospho-p38 and phospho-JNK) was similar between newborns and adults [83]. In contrast, treatment of preterm neonatal monocytes demonstrated decreased SA lipoteichoic-acid- (LTA-) induced/TLR-mediated phosphorylation of p38 and ERK in whole blood [64]. LTA-induced production of IL-1*β*, IL-6, and IL-8 increased with gestational age [64].

*Staphylococci *spp. evade clearance by the immune system in part by generating adenosine, an endogenous purine metabolite that acts via cognate seven-transmembrane receptors to induce immunomodulatory intracellular cyclic adenosine monophosphate (cAMP; Figure 1), and therefore modulate the immune response [84]. Among the effects of adenosine is to boost production of IL-6, which can inhibit neutrophil migration [41, 42, 85–88] while inhibiting production of TNF important to neutrophil activation [38, 89–91]. Neonatal mononuclear cells are particularly sensitive to the effects of adenosine [38]. Accordingly, this adenosine generating effect of *Staphylococci *spp. may be particularly effective at disarming neonatal innate defense.

*Opsonophagocytic Mechanisms*. Human newborn and adult monocytes demonstrate similar phagocytic capacity and intracellular killing of SE [83]. However, preterm neonatal neutrophils demonstrate impaired SE-induced neutrophil oxidative burst compared to term newborns [57]. The plasma of premature neonates, especially extremely low birth weight (ELBW) newborns, had a lower opsonophagocytic capacity than term neonates and adults for SA [65].

The impact of these differences on the innate immune responses depending on age to SE and SA is that lower gestational age has a significant impact on the susceptibility of the individual to infection (Table 1). Since neonates have impaired sepsis-induced cytokine production, replenishing cytokines or APPs in neonates may be particularly helpful in the treatment of the preterm newborn. Knowing the deficiencies in the innate immune response may provide specific avenues for developing new treatments. 

## 6. Potential Therapeutics

Although SE infections are often cleared from the newborn bloodstream within a few days of intravenous antibiotics (e.g., vancomycin), these infections can recur and are associated with substantial morbidity and healthcare costs [92–94]. Moreover, vancomycin resistance may be emerging [95]. Accordingly, additional preventative and therapeutic strategies are needed.

Injection of the *S. simulans*-derived metalloendopeptidase lysostaphin that cleaves crosslinking pentaglycine bridges in staphylococcal cell walls to MRSA-infected 2-day-old mice reduced bacterial load, improved neonatal weight gain, and enhanced survival similarly to vancomycin [96].

Another approach to addressing staphylococcal infection is to boost host defense by enhancing the quality of phagocytic responses in early life. In a study examining leukocytes from extremely premature infants (24–32 weeks of gestation), treating their leukocytes *ex vivo* with interferon (IFN)-*γ* reversed their innate immune deficiency [65]. Plasma from whole blood of ELBW newborns treated with IFN-*γ* significantly increased the phagocytosis of SA and SE by HL-60 cells [65]. This suggests that further studies are warranted to explore any potential therapeutic benefits for newborns. Administration of granulocyte-macrophage colony-stimulating factor (GM-CSF) to human newborns increased neutrophil production but had no impact on sepsis [97]. Treating septic very low birth weight infants with granulocyte (G)-CSF increased neutrophil phagocytic activity and oxidative burst but had no reported impact on sepsis due to the low number of sepsis patients in the study [98].

Since newborns have an increased susceptibility to sepsis, treating newborns with antibodies specific for SE and/or SA was examined. However, giving immunoglobulin intravenously from donors that had high titers of antibodies to SE and/or SA failed to significantly impact sepsis in preterm newborns [99–101]. However, the authors report a trend towards a decreased incidence ratio for SA infection in patients treated with antistaphylococcal antibodies suggesting that a higher-powered study would be required to examine efficacy more accurately [101].

## 7. Future Directions/Prospects

Many studies have documented late-onset sepsis in neonates due to staphylococcal infection. Current knowledge of the distinct immune system of preterm newborns provides at least three approaches to prevent and/or treat *Staphylococcus *spp. infections.

1. *PRR Activation to Enhance Innate Antibacterial Defense.* Activation of PRRs can change the set point of the innate immune system resulting in enhanced host defense in response to subsequent challenge with a range of pathogens. This phenomenon is a form of innate memory, that is, demonstrable in many life forms, including plants and insects and has been called “trained immunity” [102]. For example, intraperitoneal administration of a TLR agonist 24 hours prior to a polymicrobial peritonitis challenge markedly enhances neonatal defense and survival after subsequent polymicrobial sepsis by boosting bacteria-induced cytokine production and phagocytic function [103].
2. *Use of TLR Antagonists as Adjunctive Anti-infective Therapy*. In contrast to preexposure to TLR agonists to boost innate defense prior to an infection, a different strategy may be beneficial during an established infection. Antibiotic-killed bacteria are no longer viable but do continue to activate PRRs thereby inducing inflammation that can be harmful to multiple organ systems, including the brain [27]. Accordingly, adjunctive treatment with a TLR antagonist together with conventional antibiotics may help resolve infection-associated inflammation and reduce consequent morbidity of infection as has been demonstrated *in vivo* in other models and clinical settings [104, 105].
3. *Use of APPs as Novel Anti-infective Agents.* APPs with activity against Gram-positive bacteria include defensins, cathelicidins, lactoferrin and secretory phospholipase A2 [106, 107]. Biopharmaceutical development of APPs as novel anti-infective agents is proceeding, and replenishing deficient levels in preterm newborns either by direct infusion of APPs or by administration of agents that induce their expression may represent a promising approach to reduce infection.

Overall, further research on unique aspects of the neonatal host/staphylococcal pathogen interaction is warranted to assess the safety and efficacy of the aforementioned approaches and to identify new ones. 

## 8. Discussion

This review has summarized recent studies of the innate immune response in preterm and full-term neonates compared to adults in response to SE or SA infection. We highlight important progress in defining the distinct innate immune response of newborns to *Staphylococci *spp. As there are currently limited strategies to address disease caused by these pathogens, it is hoped that recent progress in defining relevant host defense and pathogenic factors [108, 109] will inform new approaches to prevent and treat late onset sepsis due to *Staphylococci *spp. 

## Figures and Tables

**Table 1:** Differences in the innate immune response between preterm newborns, full-term newborns, infants, and adults in response to SE and SA.

	Preterm newborns	Full-term newborns	Adults
Monocyte TLR2 expression	+	+	+
Monocyte MyD88 expression	?	+	++
Phosphorylation of signaling molecules in response to G+	+	++	++
Th1 cytokine expression	+	++	+++
Neutrophil oxidative burst	+	++	++
Plasma opsonophagocytic capacity	+	++	++
Plasma antimicrobial proteins and peptides	+	++	+++
